# Flexible and Accurate Simulation of Radiation Cooling with FETD Method

**DOI:** 10.1038/s41598-018-21020-w

**Published:** 2018-02-08

**Authors:** Huan Huan Zhang, Wei E. I. Sha, Zhi Xiang Huang, Guang Ming Shi

**Affiliations:** 1 School of Electronic Engineering, Xidian University, Xi’an, 710071 China; 2 College of Information Science and Electronic Engineering, Zhejiang University, Hangzhou, 310058 China; 3 Key Laboratory of Intelligent Computing and Signal Processing, Ministry of Education, Anhui University, Hefei, 230601 China; 4 State Key Laboratory of Milimeter Waves, Southeast University, Nanjing, 210096 China

## Abstract

Thermal management and simulation are becoming increasingly important in many areas of engineering applications. There are three cooling routes for thermal management, namely thermal conduction, thermal convection and thermal radiation, among which the first two approaches have been widely studied and applied, while the radiation cooling has not yet attracted much attention in terrestrial environment because it usually contributes less to the total amount of thermal dissipation. Thus the simulation method for radiation cooling was also seldom noticed. The traditional way to simulate the radiation cooling is to solve the thermal conduction equation with an approximate radiation boundary condition, which neglects the wavelength and angular dependence of the emissivity of the object surface. In this paper, we combine the heat conduction equation with a rigorous radiation boundary condition discretized by the finite-element time-domain method to simulate the radiation cooling accurately and flexibly. Numerical results are given to demonstrate the accuracy, flexibilities and potential applications of the proposed method. The proposed numerical model can provide a powerful tool to gain deep physical insight and optimize the physical design of radiation cooling.

## Introduction

Cooling techniques have become increasingly important in many areas. For instance, with the broad applications of three-dimensional (3-D) packaging and interconnection technologies, the integrated circuits (ICs) possess great benefits including high density, reduced signal delay, low power consumption, capable of heterogeneous integration, etc. But meanwhile it also jeopardizes the performance and reliability of ICs due to the increased power densities and high on-chip temperature^1–3^. Research indicates that more than 50% of ICs failure is associated with the thermal issues^4^. Driven by the modern military requirements, the power level of the RF/microwave components becomes higher and higher, leading to extremely high operating temperature^5^. Therefore, the effective dissipation of the high heat flux is indispensable for the normal operation of these components. Moreover, in the field of photovoltaics, the increased temperature of the solar cells induced by the sunlight shows a great influence on its reliability and efficiency. Each increase in temperature of 1 °C results in a relative efficiency drop of about 0.45% for crystalline silicon solar cells^6^. Countless examples can be provided to prove the necessity of developing cooling techniques.

Since the heat transfer mechanisms can be categorized into three different modes: conduction, convection and radiation, the corresponding cooling techniques also include conduction cooling, convection cooling and radiation cooling^7^. Among them the first two cooling ways have been widely studied and applied. For example, the heat flux in ICs is usually conducted to the heat sink through interconnects and thermal vias, then dissipated to the air through convective heat transfer between the air and the surface of the heat sink. The radiation cooling technique was less utilized in terrestrial environment because it is considered to contribute less to the total amount of thermal dissipation when it is combined with other cooling techniques. However, recent research has shown that theoretically large temperature reduction as much as 60 °C can be obtained by adopting a selective thermal emitter and decreasing the parasitic thermal load simultaneously^8,9^.

Nowadays, the numerical simulation methods for thermal management and cooling designs are applied more and more extensively. Similar to the applications of the three cooling techniques, the numerical methods of the conduction and convection cooling are also well developed compared to those of radiation cooling. Commonly adopted numerical methods for the transient thermal analysis include integral equation-based method, finite-difference time-domain (FDTD) method and finite-element time-domain (FETD) method^10–22^, etc. The integral equation-based method is very suitable for open problems due to the involvement of Green’s function. But its level of complexity will increase enormously when dealing with inhomogeneous material. The differential equation-based methods, such as FDTD and FETD, are preferred choice for analyzing complex inhomogeneous material in bounded geometries. The FDTD method is widely applied due to its simplicity and computational efficiency. Since orthogonal grids are employed in the conventional FDTD method, leading to the staircase error. Consequently, the mesh density must be increased dramatically to model objects with curved surfaces. By contrast, the FETD method with tetrahedral elements is more flexible to model arbitrarily-shaped geometries. Most of the aforementioned studies on numerical methods focus on the simulation of conduction cooling and convection cooling. Very few of them involve the simulation of radiation cooling. In particular, the radiation boundary condition used in these studies is an approximate one which neglects the wavelength and angular dependence of the emissivity of the object surface. For the real-world applications, this emissivity is always a function of the wavelength and direction angle. In this paper, the heat conduction equation with a rigorous radiation boundary condition is discretized with the FETD method to simulate the radiation cooling phenomena accurately and flexibly.

## Methods

### Governing Equation and Its Definite Conditions

**Figure 1:**
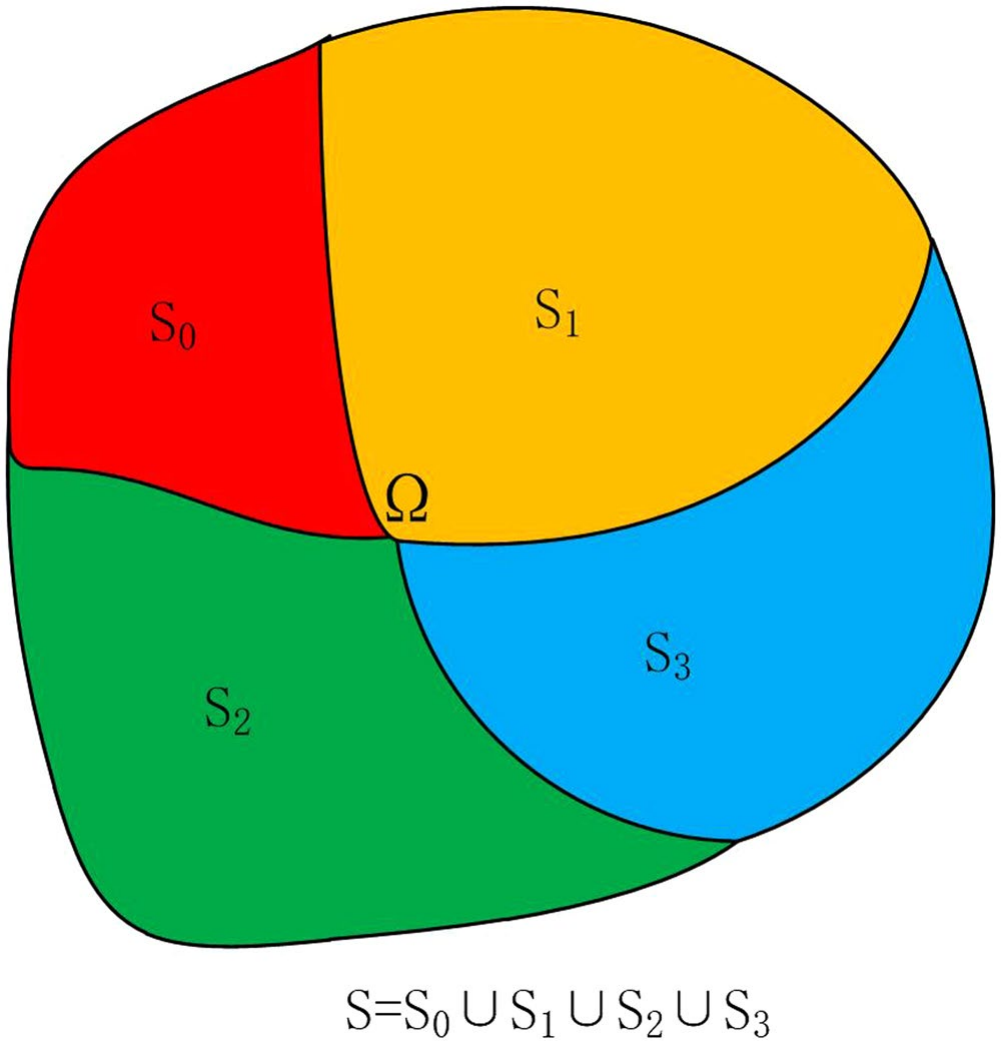
An arbitrarily shaped object Ω and its boundary surface *S* = *S*_0_ ∪ *S*_1_ ∪ *S*_2_ ∪ *S*_3_.

As shown in Fig. 1, consider an arbitrarily shaped object Ω within which there is no bulk motion, the governing equation for its transient thermal analysis can be expressed as

1 $$\rho {c}_{\rho }\frac{\partial T}{\partial t}=\nabla \cdot (\kappa \nabla T)+Q$$

where *ρ* is the density of the materials, *c*_*ρ*_ is the specific heat capacity, *T* denotes the temperature distribution as a function of time and space, *κ* is the thermal conductivity, and *Q* represents the heat source, corresponding to the energy generated per unit volume in the medium.

The differential Eq. (1) with predefined boundary condition and initial condition can be solved to obtain the spatial temperature distribution in the medium. There are four categories of commonly-used boundary conditions in thermal analyses. For the convenience of description, the surface of Ω is denoted by *S* = *S*_0_ ∪ *S*_1_ ∪ *S*_2_ ∪ *S*_3_. If *S*_0_ is maintained at a fixed temperature *T*_*s*_, the Dirichlet boundary condition will be applied:

2 $$T={T}_{s},\quad {\bf{r}}\in {S}_{0}$$

The second category of boundary condition is termed as the Neumann condition, corresponding to a fixed heat flux at the surface *S*_1_:

3 $$-\hat{n}\cdot \kappa \nabla T={q}_{s},\quad {\bf{r}}\in {S}_{1}$$

where $\hat{n}$ denotes the unit outward normal vector of the boundary surface. The surface *S*_1_ becomes a perfectly insulated or adiabatic surface when *q*_*s*_ = 0.

The third category of boundary condition is the convection boundary condition obtained from the Newton’s law of cooling:

4 $$-\hat{n}\cdot \kappa \nabla T=h\,(T-{T}_{sur}),\quad {\bf{r}}\in {S}_{2}$$

where *h* is the convective heat transfer coefficient. *T*_*sur*_ refers to the surrounding temperature. This boundary condition is utilized to describe the heat transfer between the surface *S*_2_ and the fluid moving over the surface.

**Figure 2:**
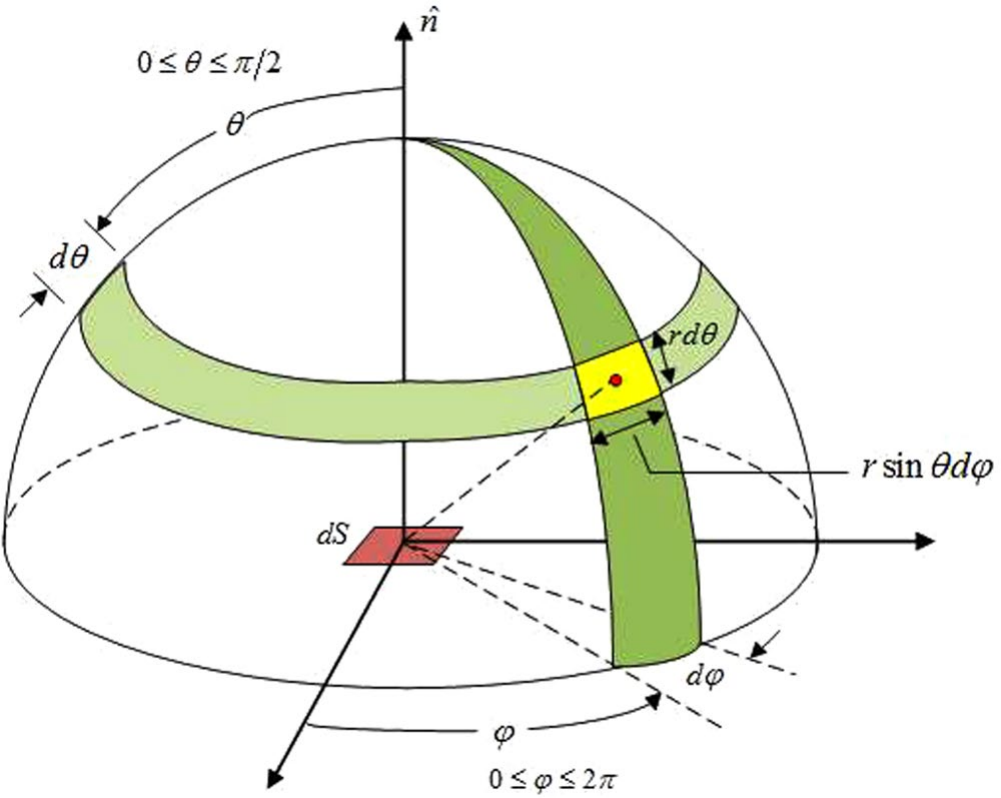
Illustration of the zenith angle and azimuthal angle in the spherical coordinate system.

If the temperature of the medium is above absolute zero, it will radiatively emit heat to outer space, which is called thermal radiation. The fourth category of boundary condition, namely radiation boundary condition, will be applied if thermal radiation occurs between the surface *S*_3_ of the medium and its surrounding environment:

5 $$-\hat{n}\cdot \kappa \nabla T={P}_{rad}\,(T)-{P}_{atm}\,({T}_{sur}),\quad {\bf{r}}\in {S}_{3}$$

6 $${P}_{rad}\,(T)={\int }_{0}^{\infty }{\int }_{0}^{2\pi }{\int }_{0}^{\frac{\pi }{2}}{I}_{\lambda ,B}\,(\lambda ,T)\,\varepsilon \,(\lambda ,\theta ,\phi )\,\cos \,(\theta )\,\sin \,(\theta )\,d\theta d\phi d\lambda $$

7 $${P}_{atm}\,({T}_{sur})={\int }_{0}^{\infty }{\int }_{0}^{2\pi }{\int }_{0}^{\frac{\pi }{2}}{I}_{\lambda ,B}\,(\lambda ,{T}_{sur})\,\varepsilon \,(\lambda ,\theta ,\phi )\cos \,(\theta )\,\sin \,(\theta )\,d\theta d\phi d\lambda $$

where *P*_*rad*_(*T*) denotes the power radiated by the structure per unit area. *P*_*atm*_(*T*_*sur*_) represents the power absorbed from the surrounding atmosphere per unit area. *ε*(*λ*, *θ*, *φ*) is the spectral and directional emissivity of *S*_3_. As shown in Fig. 2, *θ* is the zenith angle, while *φ* is the azimuthal angle. *λ* denotes the wavelength. *I*_*λ*,*B*_(*λ*, *T*) refers to the spectral radiance of a blackbody at temperature *T*.

8 $${I}_{\lambda ,B}\,(\lambda ,T)=\frac{2h{c}_{0}^{2}}{{\lambda }^{5}}\frac{1}{\exp (\frac{h{c}_{0}}{\lambda {k}_{B}T})-1}$$

where *c*_0_ = 2.998 × 10^8^ *m*/*s* is the speed of light in free space. *k*_*B*_ = 1.381 × 10^−23^ *J*/*K* is the Boltzmann constant. *h* = 6.626 × 10^−34^ *J* · *K* is the Planck constant.

Equations (6) and (7) are the rigorous expression for *P*_*rad*_(*T*) and *P*_*atm*_(*T*_*sur*_). In the traditional radiation boundary condition, the emissivity of the medium surface is assumed to be independent of the wavelength and direction, namely *ε*(*λ*, *θ*, *φ*) = *ε*_0_. Since the total emissive power of a blackbody can be calculated as

9 $${\int }_{0}^{\infty }{\int }_{0}^{2\pi }{\int }_{0}^{\frac{\pi }{2}}{I}_{\lambda ,B}\,(\lambda ,T)\cos \,(\theta )\,\sin \,(\theta )\,d\theta d\phi d\lambda =\sigma {T}^{4}$$

where *σ* = 5.68 × 10^−8^*W*/*m*^2^*K*^4^ is the Stefan–Boltzmann constant. We can obtain the traditional radiation boundary condition as

10 $$-\hat{n}\cdot \kappa \nabla T={\varepsilon }_{0}\sigma ({T}^{4}-{T}_{sur}^{4}),\quad {\bf{r}}\in {S}_{3}$$

When adopting the rigorous expression of radiation boundary condition in (5), the trapezoidal rule of numerical integration is utilized to calculate (6) an (7)^23^. It is worth mentioning that the infinite integral over the wavelength should be truncated into a finite one. Equation (9) can be used to test the accuracy of different truncation boundary and resolution. Numerically, we find that 0.4% relative accuracy can be achieved with a spectral resolution of 2 *nm* from 1 *μm* to 1000 *μm* and with 3 degree angular resolution.

In Cartesian coordinates, the initial condition can be specified as

11 $$T(x,y,z;t=0)={T}^{0}(x,y,z)$$

where *T*^0^(*x*, *y*, *z*) is the temperature distribution at *t* = 0.

### Finite-Element Time-Domain Method

The FETD method is employed to solve Eq. (1) with the above mentioned boundary conditions and the initial condition for obtaining the time-varying temperature distribution of the medium. Firstly, the medium Ω is discretized into a set of tetrahedral elements Ω_*i*_. Nodal basis functions are employed to expand *T* in each element Ω_*i*_^24^. Then considering the aforementioned Neumann, convection and radiation boundary conditions simultaneously, we can obtain the spatially discrete form of equation (1) by using the Galerkin’s scheme:

12 $$[C]\{\frac{\partial T}{\partial t}\}+[K]\{T\}=\{f(T)\}$$

where

13 $${[C]}_{ij}=\rho {c}_{\rho }{\int }_{V}{N}_{i}{N}_{j}dV$$

14 $${[K]}_{ij}=\kappa {\int }_{V}\nabla {N}_{i}\cdot \nabla {N}_{j}dV+h{\int }_{{S}_{2}}{N}_{i}{N}_{j}dS$$

15 $${\{f(T)\}}_{i}={\int }_{V}{N}_{i}QdV-{\int }_{{S}_{1}}{N}_{i}{q}_{s}dS+h{\int }_{{S}_{2}}{N}_{i}{T}_{sur}dS-{\int }_{{S}_{3}}{N}_{i}({P}_{rad}(T)-{P}_{atm}({T}_{sur}))dS$$

*N*_*i*_ and *N*_*j*_ refer to the *i*-th and *j*-th nodal basis functions, respectively. {*T*} refers to the time-dependent expansion coefficients to be solved, which is also the vector constituted by the temperature at the nodes of the elements. For the nodes residing on *S*_0_, we need to impose the Dirichlet boundary condition. The corresponding unknown expansion coefficients on *S*_0_ are explicitly given by (2), only the unknown coefficients not over *S*_0_ are to be solved by (12).

Suppose that the temperature of the nodes at time *t*_*i*_ = *i*Δ*t* is {*T*_*i*_}. Δ*t* is the time step size. The Crank-Nicolson scheme is adopted for the temporal discretization of (12) to arrive at an unconditionally stable system^25^:

16 $$([C]+[K]\frac{{\rm{\Delta }}t}{2})\{{T}_{i}\}=([C]-[K]\frac{{\rm{\Delta }}t}{2})\{{T}_{i-1}\}+\{f({T}_{i})\}{\rm{\Delta }}t$$

Note that (16) is a nonlinear system of equations. Particularly, the last term arising from the radiation boundary condition (5) requires to compute the radiation power *P*_*rad*_(*T*) at the time *t* = *t*_*i*_. In order to avoid solving nonlinear equation, Eq. (16) at the time *t* = *t*_*i*_ is solved in an iterative manner:

17 $$([C]+[K]\frac{{\rm{\Delta }}t}{2})\{{T}_{i}^{(n+1)}\}=([C]-[K]\frac{{\rm{\Delta }}t}{2})\{{T}_{i-1}\}+\{f({T}_{i}^{(n)})\}{\rm{\Delta }}t$$

**Figure 3:**
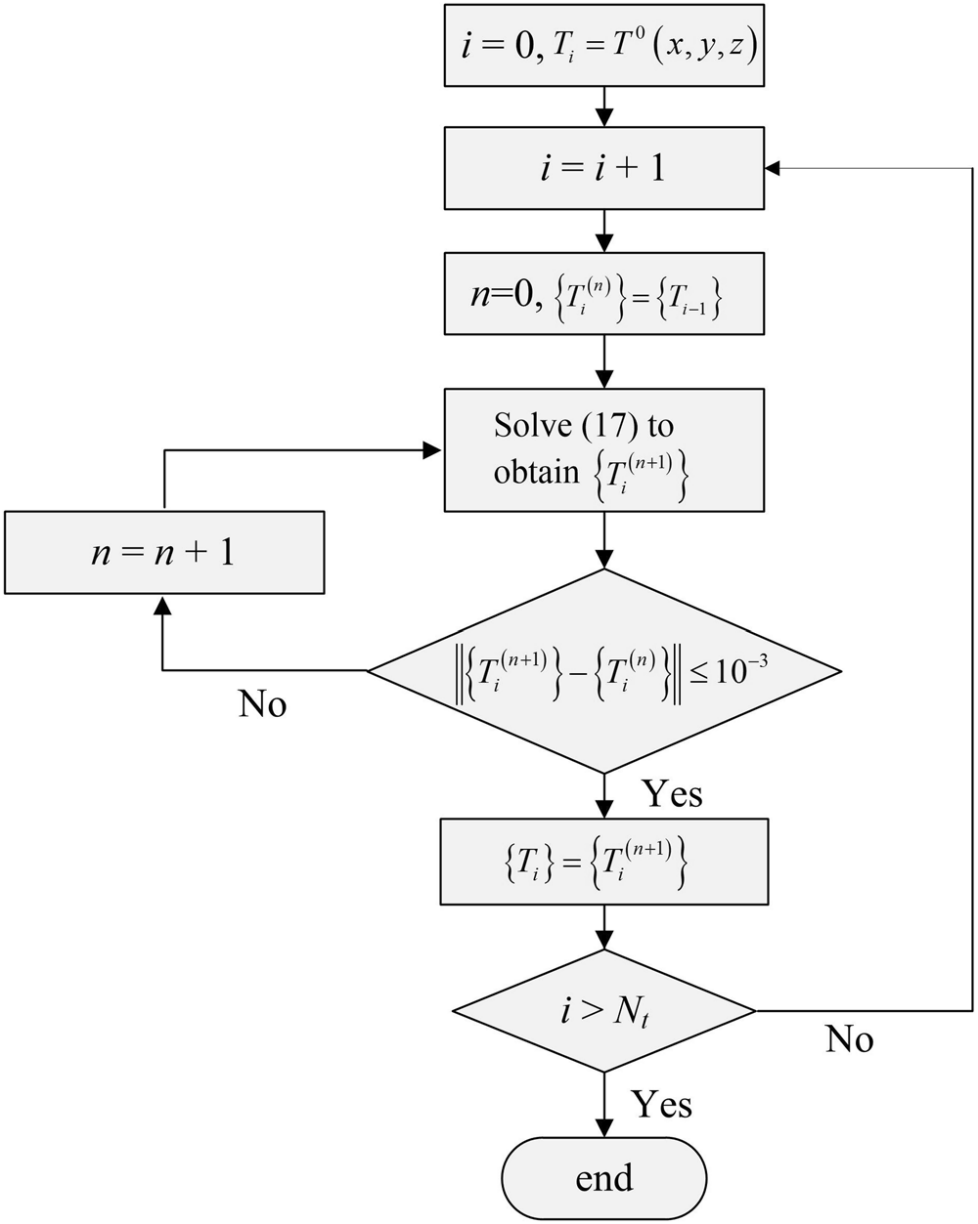
Flow diagram for solving the temperature distribution at each time step.

The initial value of $\{{T}_{i}^{(n)}\}$ is set to be $\{{T}_{i}^{\mathrm{(0)}}\}={T}_{i-1}$ in (17), which provides the values for the right hand side of (17), leaving the temperature vector $\{{T}_{i}^{(n+\mathrm{1)}}\}$ as unknowns. Then $\{{T}_{i}^{(n+\mathrm{1)}}\}$ can be solved by using the multifrontal method for sparse linear equations^26^. This process is repeated until the difference between $\{{T}_{i}^{(n+\mathrm{1)}}\}$ and $\{{T}_{i}^{(n)}\}$ is less than an acceptable tolerance. It is set to be 10^−5^ in this paper. Finally, we can obtain $\{{T}_{i}\}=\{{T}_{i}^{(n+\mathrm{1)}}\}$. Figure 3 shows the schematic picture for the numerical solution of the temperature distribution at each time step.

## Results

### Silicon Cube

**Figure 4:**
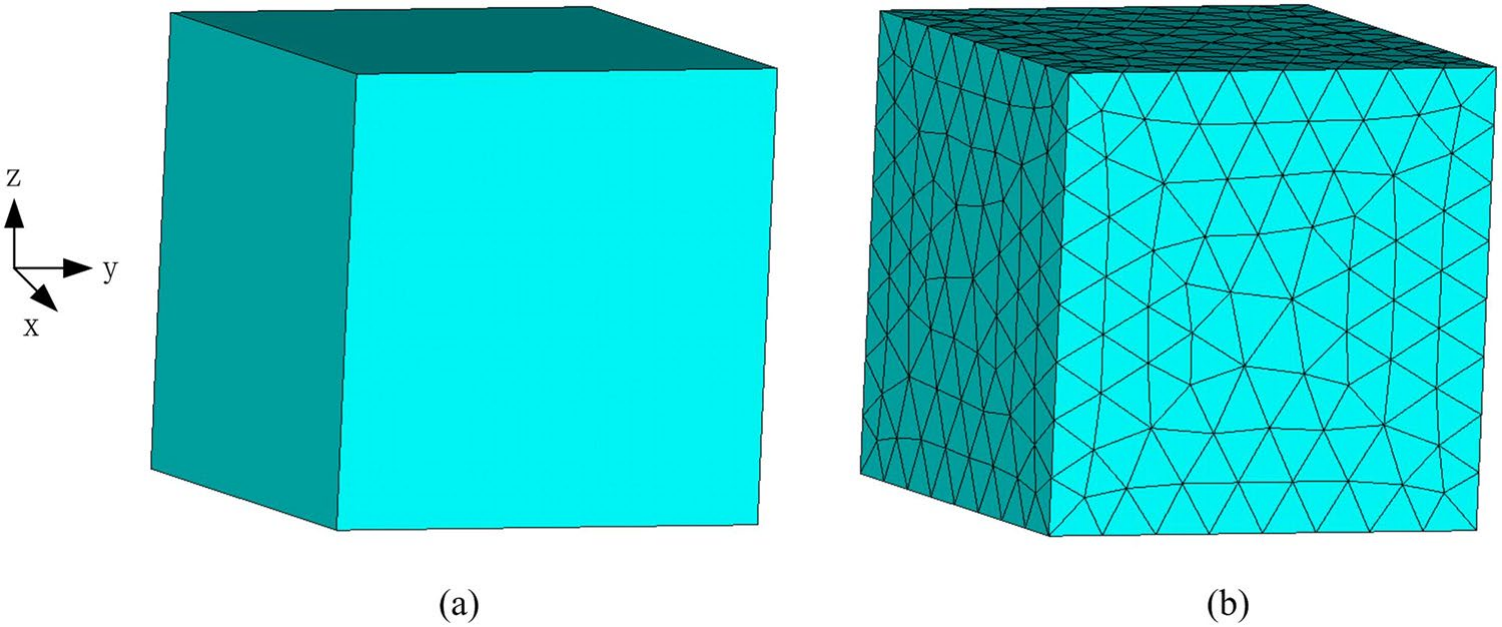
(**a**) Silicon cube model, (**b**) Meshed model with tetrahedral elements.

As shown in Fig. 4(a), a silicon cube with the side length of 0.5 *m* is studied to validate the accuracy and flexibility of the proposed method. The material parameters of the cube are *κ* = 135*W*/[*m* · *K*], *ρ* = 2330 *kg*/[*m*^3^], *c*_*ρ*_ = 704 *J*/[*kg* · *K*]. The ambient temperature is set to be 300 *K*. The initial temperature of the cube is 800 *K*. The cube is meshed into 3736 tetrahedral elements as shown in Fig. 4(b). The temperature at a randomly chosen observation point *r* = (0.185 *m*, 0.18 *m*, 0.256 *m*) is recorded in the following simulation.

**Figure 5:**
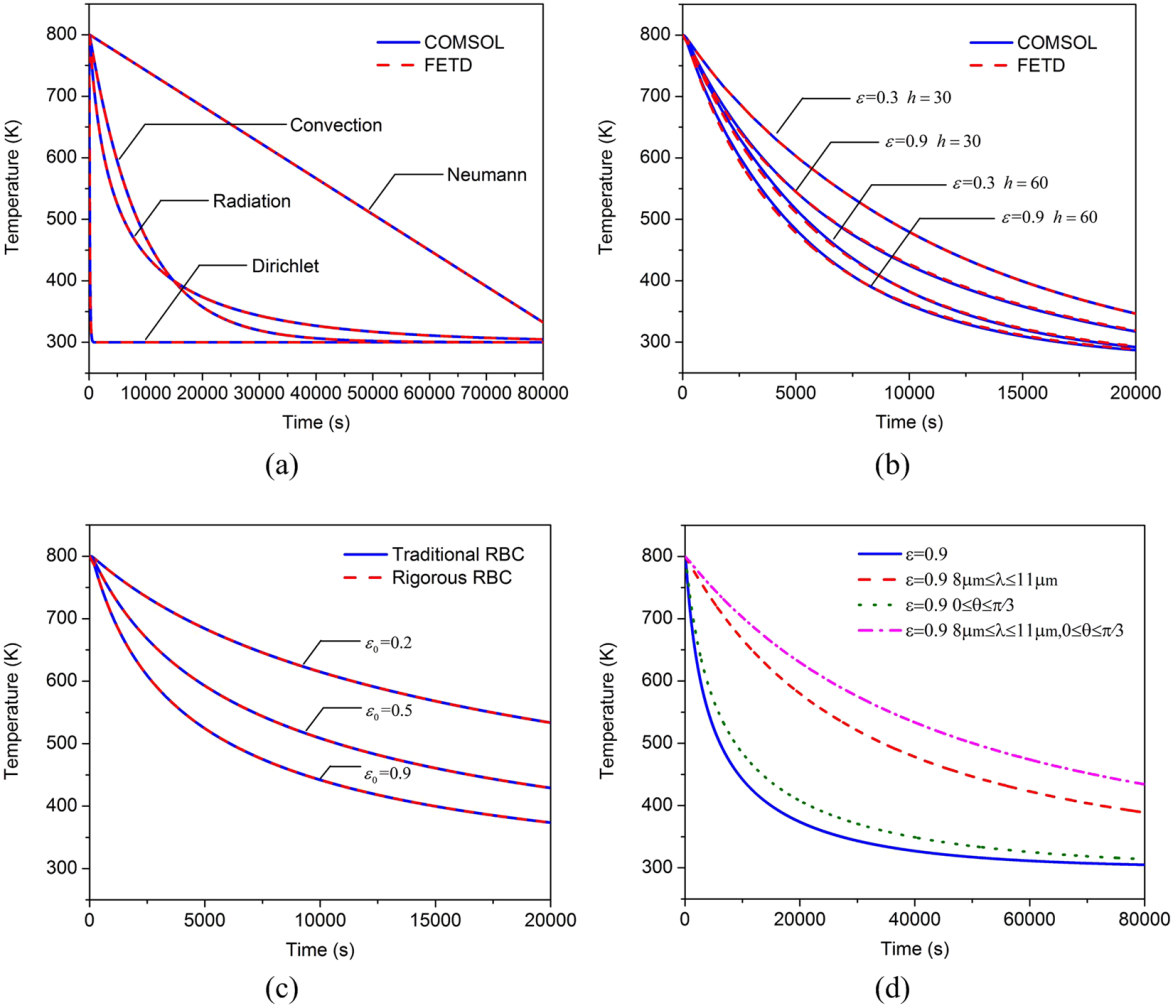
The temperature at the observation point obtained by (**a**) the proposed method and the COMSOL software with the Dirichlet, Neumann, convection and traditional radiation boundary conditions imposed to the whole boundary surface of the cube, (**b**) the proposed method and the COMSOL software with the Neumann boundary condition applied to the surfaces at x = 0 and x = 0.5, convection boundary condition applied to the surfaces at y = 0 and y = 0.5, and traditional radiation boundary condition applied to the surfaces at z = 0 and z = 0.5, (**c**) the traditional radiation boundary condition and rigorous radiation boundary condition, (**d**) the rigorous radiation boundary condition with the surface emissivity depending on the wavelength and emission angle.

Firstly, the Dirichlet, Neumann, convection and traditional radiation boundary conditions are imposed to the whole boundary surface, respectively. The temperatures on the boundary surface are fixed to be 300 *K* in the Dirichlet boundary condition. The fixed heat flux in Neumann boundary condition equals to 800 *K*/*m*^2^. The convective heat transfer coefficient is set to be *h* = 15 *W*/[*m*^2^ · *K*] in the convection boundary condition. The emissivity of the boundary surface is set to be *ε*_0_ = 0.9 in the traditional radiation boundary condition. Both the proposed method and the COMSOL software are employed to analyze this problem^27^. The temporal temperature at the observation point is shown in Fig. 5(a). Very good agreement between the proposed method and the COMSOL software is achieved. We can also find that the temperature at the observation point before 14000 s decreases faster when applying the radiation boundary conditions than using the convection boundary condition, which implies that the radiation cooling can be very effective especially when the temperature of object surface is high since it is proportional to the fourth power of temperature.

Secondly, different boundary conditions are imposed to different surfaces of the cube to further check the code. The fixed outward heat flux 2000 *W*/[*m*^2^] is applied on the surfaces of x = 0 and x = 0.5. The convection boundary condition is imposed on the surfaces of y = 0 and y = 0.5. The radiation boundary condition is applied on the surfaces of z = 0 and z = 0.5. As shown in Fig. 5(b), four groups of the convective heat transfer coefficients and emissivities are tested, again good agreement between COMSOL and the proposed method can be observed. Moreover, it is easy to find that a larger convective heat transfer coefficient or emissivity can lead to faster cooling.

Thus far, we have validated the implementation of the Dirichlet, Neumann, convection and traditional radiation boundary conditions. However, the treatment of the rigorous radiation boundary condition in (5) has not been proved yet. According to (9), if we set the emissivity to be a constant *ε*_0_ independent of the wavelength and direction, the results obtained through utilizing (5) and (10) should be the same. This can be used to check the correctness of treatment of the rigorous radiation boundary condition. Figure 5(c) shows the results corresponding to *ε*_0_ = 0.2, *ε*_0_ = 0.5 and *ε*_0_ = 0.9 when applying the radiation boundary condition to the six surfaces of the cube. Obviously, the results of the rigorous radiation boundary condition agree well with those of the traditional radiation boundary condition, which demonstrates the correctness of our proposed method.

Actually, the emissivity of an object surface is seldom uniform, and it is usually dependent on the wavelength and emission angle. In order to exhibit such a feature, we consider three different kinds of emissivities: (1) The emissivity only depends on the wavelength. It equals to 0.9 in the wavelength range from 8 *μm* to 11 *μm* and is zero elsewhere. (2) The emissivity only depends on the emission angle. It equals to 0.9 in the angle range from 0 to *π*/3 and is zero in the angle range from *π*/3 to *π*/2. (3) The emissivity depends both on the wavelength and emission angle. It equals to 0.9 in the wavelength range from 8 *μm* to 11 *μm* and in the angle range from 0 to *π*/3. The emissivity is zero elsewhere. The rigorous radiation boundary condition is applied with the above three different kinds of emissivities. The temperature of the observation point can be obtained by the proposed method as shown in Fig. 5(d). Note that the case of *ε*_0_ = 0.9 in Fig. 5(c) is also replotted in Fig. 5(d) for comparison. Obviously, the cooling effect will be overestimated if adopting the traditional radiation boundary condition. It is worth mentioning that the number of iterations for the solution of (17) at each time step in these three cases are less than 5.

### 3D IC Package with a Heat Sink

**Figure 6:**
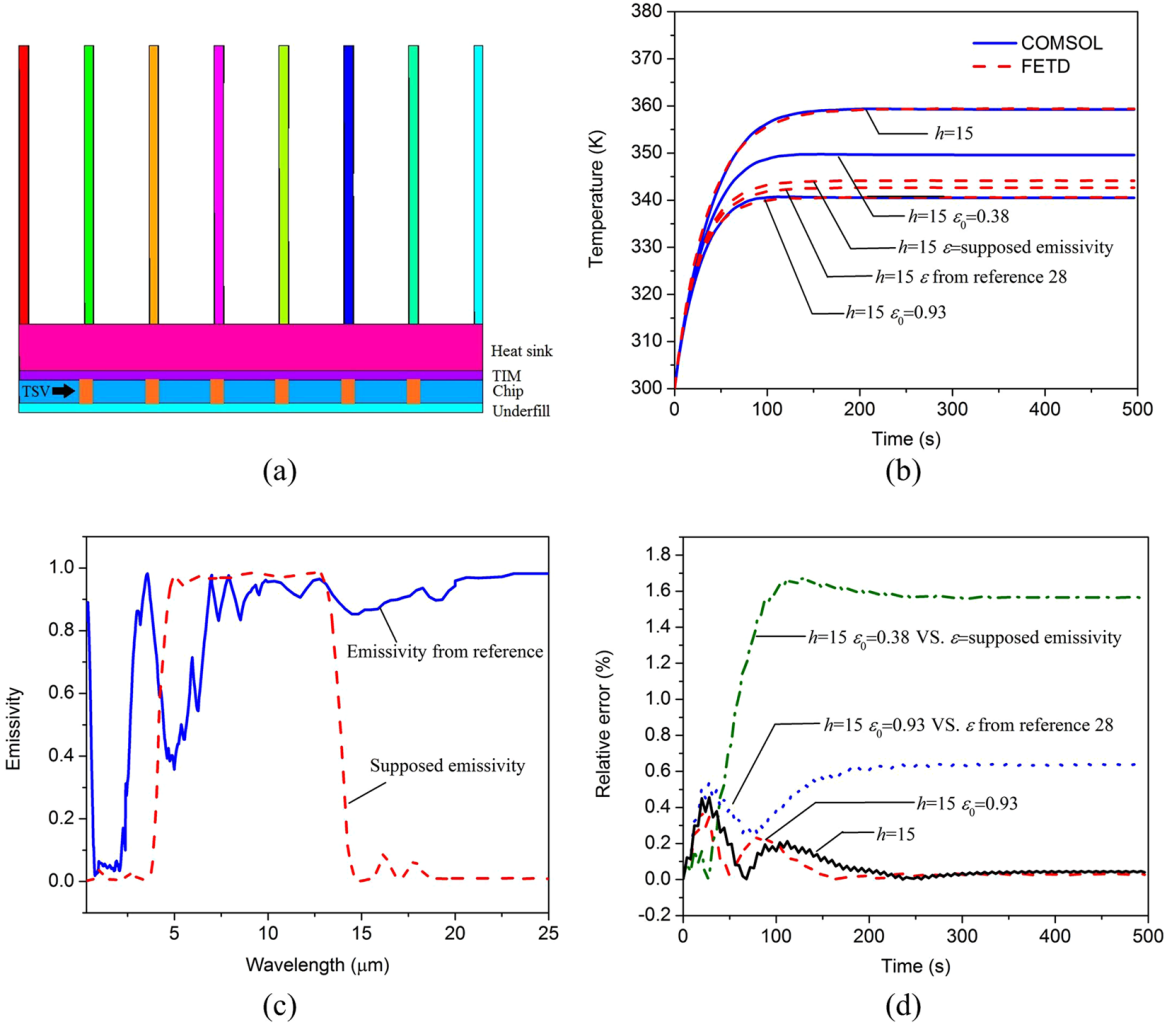
(**a**) Cross-section view of the 3D IC package model, (**b**) the temporal temperature at the observation point obtained by the proposed method and the COMSOL software, (**c**) the supposed emissivity and the emissivity in reference ^28^, (**d**) the relative errors between the proposed method and COMSOL software.

Consider a 3D IC package model with a heat sink, the cross-section view of which is shown in Fig. 6(a). The model is composed of a heat sink, thermal interface materials (TIM), a chip, 6 × 6 through-silicon-vias (TSVs) and an underfill layer. Its geometry and material parameters are summarized in Table 1. A uniform volume power of 1 *W* is assumed on the chip layer as the heat source. Initially, the system temperature is supposed to be room temperature of 300 *K*. To perform the numerical simulation, this model is discretized into 269,446 tetrahedral elements. The temperature of a randomly chosen observation point *r* = (7.25 *mm*, 0.7 *mm*, 2.875 *mm*) at the top surface of the chip is recorded.

**Table 1:** Geometry and material parameters.

Geometry	Dimension (*mm*^3^)	*κ*	*ρ*	*c*_ρ_
Heat sink	10 × 10 × 7	220	2707	896
TIM	10 × 10 × 0.2	10	2000	385
Chip	10 × 10 × 0.5	135	2330	704
TSV	0.25(Diameter) 0.5(Height)	400	8933	385
Underfill layer	10 × 10 × 0.2	50	9290	180

**Figure 7:**
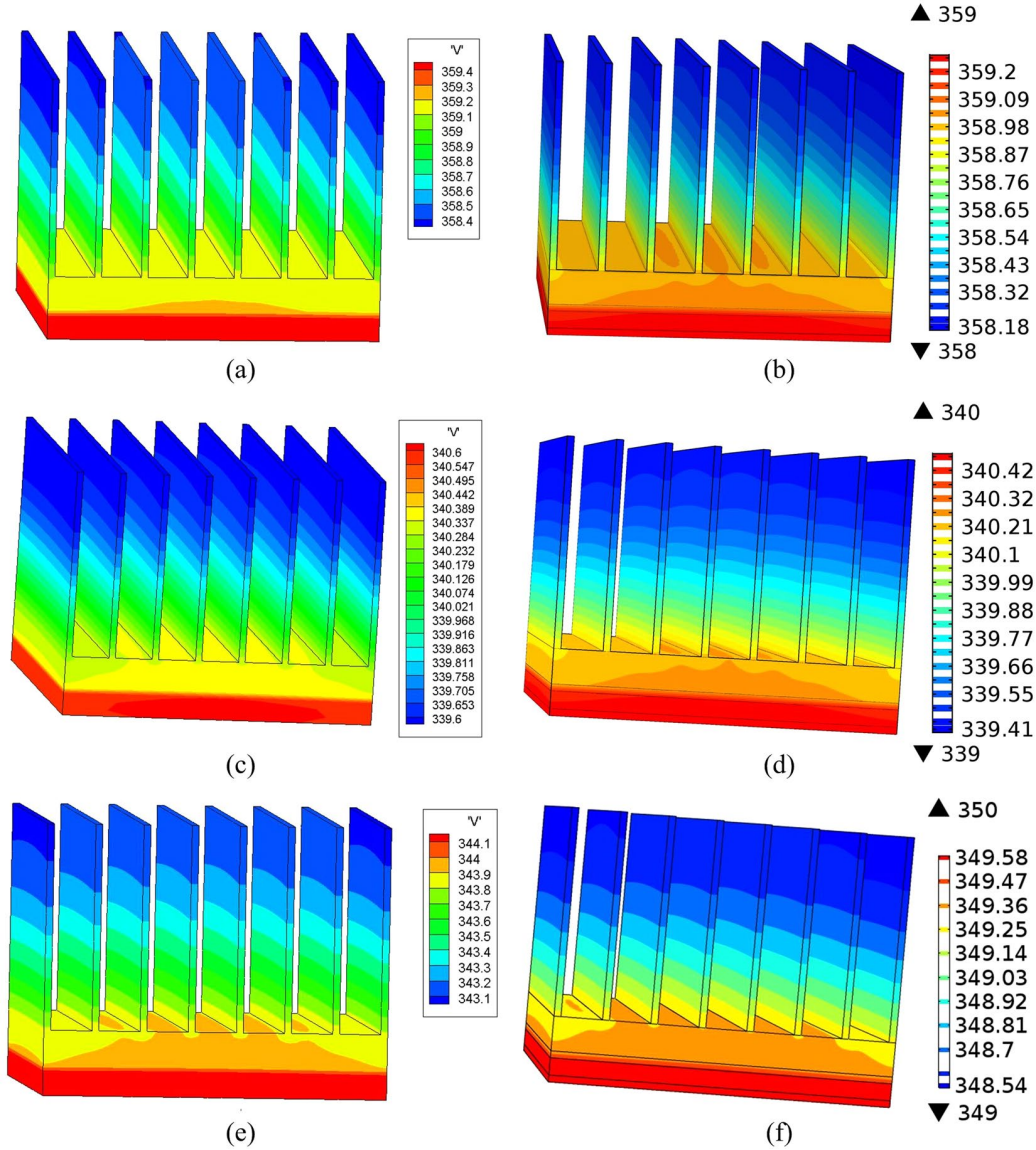
The temperature distribution of the 3D IC package model at t = 460 s obtained by (**a**) our proposed method with h = 15 *W*/[*m*^2^ · *k*], (**b**) COMSOL with h = 15 *W*/[*m*^2^ · *k*], (**c**) our proposed method with h = 15 *W*/[*m*^2^ · *k*] and *ε*_0_ = 0.93, (**d**) COMSOL with h = 15 *W*/[*m*^2^ ⋅ *k*] and *ε*_0_ = 0.93, (**e**) our proposed method with h = 15 *W*/[*m*^2^ ⋅ *k*] and *ε*_0_ = supposed emissivity, (**f**) COMSOL with h = 15 *W*/[*m*^2^ · *k*] and *ε*_0_ = 0.38.

Case 1: Firstly, the convection boundary condition is imposed on the surface of the heat sink with *h* = 15*W*/[*m*^2^ · *K*] to model the natural convection. Other surfaces are assumed to be adiabatic. Both the proposed method and the COMSOL software are employed to simulate the temperature distribution of the model. Good agreement can be observed in Figs 6(b) and 7(a,b).

Case 2: Actually, anodic oxidation treatment is usually applied to the surface of the heat sink to improve its emissivity. Thus the effect of radiative cooling can be improved. Suppose that the surface emissivity of the heat sink equals to *ε*_0_ = 0.93 after the anodic oxidation treatment. Natural convection with *h* = 15 *W*/[*m*^2^ · *K*] still exists. Then the heat conduction equation combined with the radiation boundary condition and convection boundary condition can be utilized to analyze this problem. Figures 6(b) and 7(c,d) show that the results obtained by the proposed method agree well with those by COMSOL software. The highest temperature of the observation point decreases nearly 20 °C with the radiative cooling.

Case 3: In real-world applications, the assumption of a wavelength and direction independent emissivity is often invalid. The surface emissivity usually strongly depends on the wavelength and direction, which can be measured and then used for accurate simulation of radiation cooling. Suppose that the surface of the heat sink is treated to have a measured emissivity as shown in Fig. 6(c), corresponding to an average emissivity of 0.38. The cases of supposed emissivity and average emissivity are simulated based on the rigorous radiation boundary condition and the traditional radiation boundary condition, respectively. As shown in Figs 6(b) and 7(e,f), the result of the traditional wavelength and direction independent boundary condition apparently deviates from the rigorous boundary condition. The highest temperature of the observation point decreases 10 °C with the traditional radiation boundary condition, while it decreases 16 °C with the rigorous radiation boundary condition. The significance difference suggests that a rigorous treatment of radiation boundary condition is crucial for modeling radiative cooling problems.

Case 4: Recently, a randomized, glass-polymer hybrid metamaterial was manufactured in reference^28^. The metamaterial is a 50 *μm* thick film containing 6% of microspheres by volume which has an averaged infrared emissivity 9.3. This material has a promising prospect for passive radiative cooling. Suppose the heat sink in this example is wrapped with this film and thus its surface emissivity will be improved. The measured emissivity of the film in reference^28^ is replotted in Fig. 6(c). Then the temperature distribution of the 3D IC package can be analyzed again by our proposed method. The temporal temperature of the observation point is given in Fig. 6(b). Since the measured emissivity of the film has a weak dependence on the wavelength and is very closed to the ideal wavelength and direction independent emissivity, the cooling effect with the practical emissivity is also very close to that with the ideal wavelength and direction independent emissivity.

For the aforementioned four cases, the relative errors of the temperature at the observation point between the proposed method and COMSOL software are shown in Fig. 6(d). The CPU time for the whole simulation, peak memory and average number of iterations per time step are given in Table 2. All of the numerical experiments are performed on 2.4 GHz CPU and 8 GB RAM.

**Table 2:** Computational statistics of the proposed method.

Case	1	2	3	4
CPU Time (s)	21	151	3262	4219
Memory (MB)	421	430	431	431
Average Iterations	0	6	5	6
